# Comparison of the role of vitamin D in normal organs and those affected by COVID-19

**DOI:** 10.7150/ijms.103260

**Published:** 2025-01-01

**Authors:** Rajendran Peramaiyan, Josephine Anthony, Sureka Varalakshmi, Ashok Kumar Sekar, Enas M. Ali, Al Hashedi Sallah A, Basem M. Abdallah

**Affiliations:** 1 Department of Biological Sciences, College of Science, King Faisal University, Al-Ahsa, Kingdom of Saudi Arabia.; 2 Centre of Molecular Medicine and Diagnostics (COMManD), Department of Biochemistry, Saveetha Dental College & Hospitals, Saveetha Institute of Medical and Technical Sciences, Saveetha University, Chennai 600 077, Tamil Nadu, India; 3 Department of Research, Meenakshi Academy of Higher Education and Research (Deemed to be University), Chennai - 600 078, Tamil Nadu, India.; 4 Centre for Biotechnology, Anna University, Chennai-600 025, Tamil Nadu, India.; 5 Department of Botany and Microbiology, Faculty of Science, Cairo University, Cairo 12613, Egypt.; 6 Central Laboratories, Department of microbiology, King Faisal University, 31982, Al-Ahsa, Kingdom of Saudi Arabia.

**Keywords:** post-COVID-19, COVID-19, vitamin D, immune cells, multiple organs

## Abstract

The outbreak of COVID-19 has opened up new avenues for exploring the importance of vitamin D in immunity, in addition to its role in calcium absorption. Recently, vitamin D supplementation has been found to enhance T regulatory lymphocytes, which are reduced in individuals with COVID-19. Increased risk of pneumonia and increases in inflammatory cytokines have been reported to be major threats associated with vitamin-D deficiency. Although vaccination reduces the threat of COVID-19 to a certain extent, herd immunity is the long-term solution to overcoming such diseases. Co-administration of vitamin D with certain inactivated vaccines has been reported to enhance the systemic immune response through stimulation of the production of antigen-specific mucosal immunity. COVID-19 was found to induce multiple organ damage, and vitamin D has a beneficial role in various organs, such as the intestines, pancreas, prostate, kidneys, liver, heart, brain, and immune cells. The consequences that occur after COVID-19 infection known as long COVID-19 are also a concern as they accumulate and target multiple organs, leading to immune dysregulation. The present review covers the overall role and impact of vitamin D and its deficiency for various organs in normal conditions and after COVID-19 infection, which is still a serious issue.

## Introduction

Apart from bone homeostasis, vitamin D plays a vital role in many organs and improves immunity in individuals infected with COVID-19 1, 2. Vitamin D has also been documented to prevent COVID-19-induced multiple-organ damage and long-term complications 3, 4. The risk factors and consequences vary widely between COVID-19 and long COVID-19 5. Recently, hypertension, immunosuppression, psychiatric disorders, thrombosis, and other conditions have been reported as consequences of long COVID-19 6.

Vitamin D acts as a double-edged sword for individuals with COVID-19 in that it can have both protective and harmful effects. It exhibits anti-inflammatory properties that can mitigate the severity of cytokine storms associated with severe COVID-19, but an excess of Vitamin D or its analogs can lead to hypercalcemia, which may pose additional health risks. Thus, careful management of vitamin D levels is crucial in this context. This review focuses on the detailed role of vitamin D in various organs in normal conditions and after infection with COVID-19.

Increases in biomarkers such as D-dimer, C-reactive protein, interleukin-6, and neutrophil count are risk factors during long COVID-19 7. COVID-19 survivors experiencing long COVID also exhibit lower levels of 25-hydroxyvitamin D (25(OH)D) 8. Vitamin D deficiency has been linked to a higher susceptibility to COVID-19 and increased severity of the disease 8. The D-CIMA meta-analysis indicated that individuals with low serum levels of vitamin D have 1.64-times higher risk of contracting COVID-19. Additionally, findings from the D-CSMA meta-analysis indicated that those with serum levels of 25(OH)D below 20 ng/mL or 50 nmol/L had a 2.42-times higher likelihood of experiencing severe COVID-19 9. Recent randomized controlled trials (RCTs) and non-randomized intervention studies (NRISs) have found that while vitamin D supplementation did not significantly reduce the risk of COVID-19 infection, it has protective benefits in terms of reducing mortality and the need for intensive care 10.

An umbrella meta-analysis study was conducted to assess the impact of vitamin D supplementation on clinical outcomes and mortality rates among individuals with COVID-19. The findings revealed that vitamin D levels significantly affected mortality rates, disease severity, admission to the intensive care unit (ICU), and the need for mechanical ventilation. It is very important to monitor vitamin D status in all critically ill patients, including those with COVID-19 11. Therefore, well-randomized clinical trials need to be carried out to explore the exact mechanism of vitamin D and its protective effect against prolonged COVID-19. This review discusses vital organs and the effects of COVID-19, which can lead to mortality or long-term consequences.

### Vitamin D and immune cells

The implications of vitamin D in the immune system have gained much attention as the susceptibility to infection increases in cases of deficiency 12. Vitamin D functions by binding to vitamin D receptors (VDRs), translocating to the nucleus, and regulating the transcription of vitamin-D-responsive genes. The expression of VDR occurs in immune cells such as monocytes, macrophages, dendritic cells, B cells, T cells, and antigen-presenting cells in the immune system, where it modulates the innate and adaptive immune responses 12-14. Immune cells also have the ability to synthesize the active form of vitamin D metabolites. Generally, vitamin D is found to affect both T cell activation and differentiation by decreasing the levels of IFNg and IL-17 while increasing IL-4 and IL13 levels 14-16.

Chun *et al.* documented the response of macrophages and dendritic cells to the active and circulating vitamin D metabolite, 25(OH)D 17. The first large-scale genomic studies showed that vitamin D response elements (VDREs) occur within the gene promoters of two antibacterial proteins: cathelicidin and β-defensin 2 (DEFB4) 18,19. The study also reported a simultaneous role of nuclear factor-κB (NF-κB) response elements within the DEFB4 gene promoter. The impact of cathelicidin plays a role upon vitamin-D3 muramyl dipeptide (MDP) co-treatment, which demonstrates a possible role of NF-κB and VDR in various immunomodulatory functions.

#### Role of vitamin D role in immune cells and COVID-19

A recent case study by Kongsbak-Wismann *et al.* demonstrated the vital role of vitamin D in boosting the adaptive immune response against the COVID-19 virus 20. Individuals with COVID-19 display reduced expression of the VDR in peripheral blood cells compared to controls, particularly among males 21. Al-Jaberi *et al.* identified a novel mutation in the VDR in an individual with hereditary vitamin-D-resistant rickets (HVDRR), which is an autosomal recessive disease 22. The mutation associated with VDR was reported in the DNA-binding domain, which inhibited the transcriptional activity of the vitamin-D-VDR complex.

However, the parents of the patient with HVDRR were heterozygous for that particular VDR gene, so their T-cells elicited a significant reduction in vitamin-D responsiveness. Interestingly, both parents and the patient were infected with COVID-19, thus providing an opportunity to examine the role of vitamin D signaling in the immune response. On the other hand, an investigation of COVID-19 progression and the immune response within a family with a mutated, non-functional VDRs showed that vitamin D signaling was not essential for the family members to develop a robust adaptive immune response to SARS-CoV-2 23. The activation, differentiation, and generation of CD4+ and CD8+ SARSCoV-2-specific T cells could happen in the absence of vitamin D signaling 24. Vitamin D is reported to reduce the elevated levels of cytokines in individuals with severe COVID-19 and thus plays a pivotal role in innate immunity against the disease, like the generation of cathelicidin 25.

**Figure 1:**
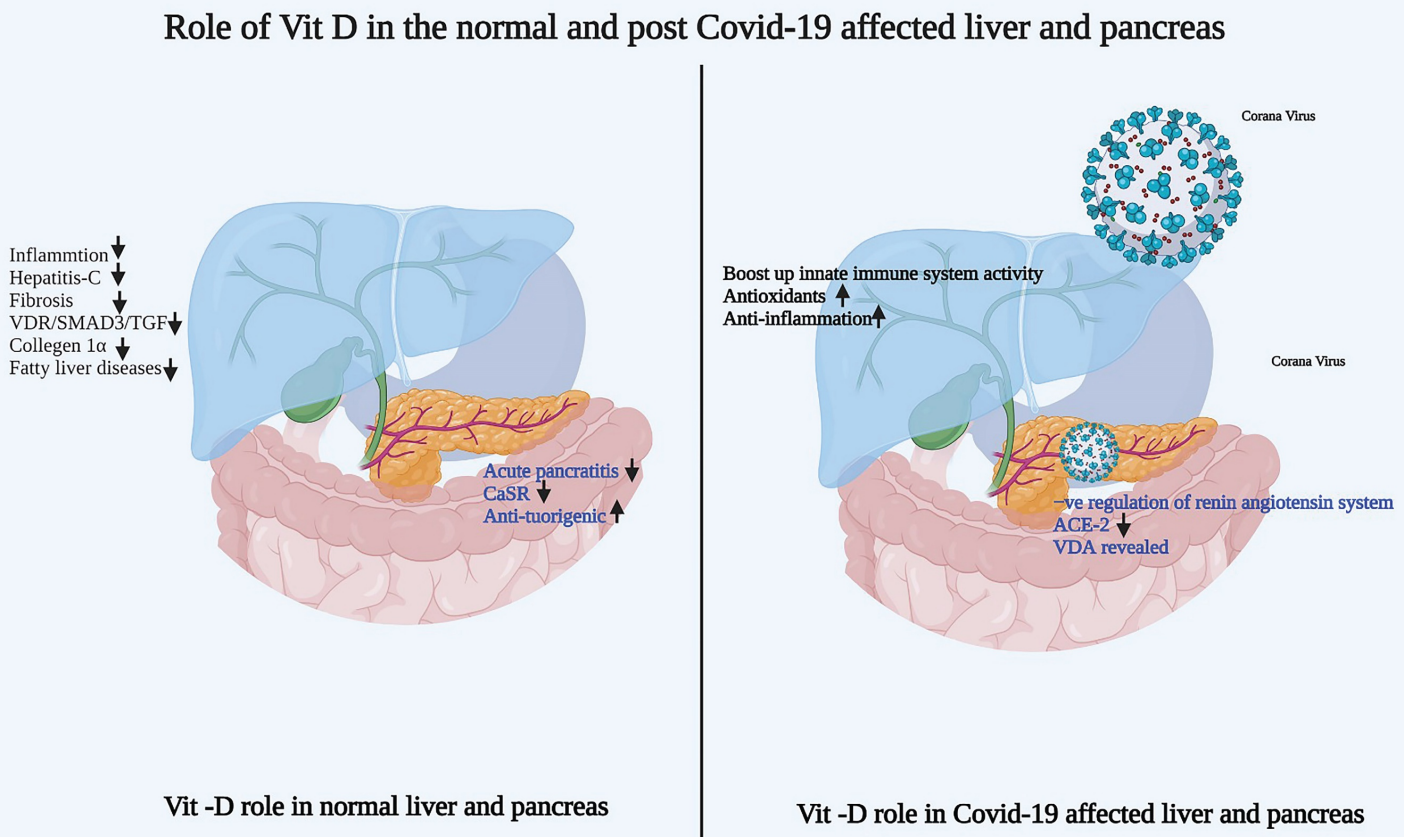
Role of vitamin-D in normal and post COVID-19 affected patient's immune cells. a) Vitamin-D role in normal immune cells b) Vitamin-D role in post COVID-19 affected patient's immune cells. Image created from Bioreder.com.

Significant variation has been observed between male and female individuals with COVID-19 in terms of mortality. The mortality rate is higher among males than females, which is likely due to the presence of androgens, which are correlated with lower efficiency in eliciting an adaptive immune response. Female individuals also have enhanced adaptive immunity due to the presence of estrogens and a higher level of IgG antibodies 26. Human β-defensins and cathelicidin are the primary lines of defense against bacterial and viral infections as they have antimicrobial activity and promote innate and adaptive immunity against bacteria and viruses. Ghelani *et al.* reported consolidated research related to the role of vitamin D in the treatment and inhibition of COVID-19 27. The role of vitamin D in normal immune cells and immune cells after COVID-19 infection is shown in Figure 1.

### Vitamin D and the intestines

Vitamin D deficiency was reported to be associated with inflammatory bowel disease followed by malignant transformation 28. Crohn's disease and ulcerative colitis are highly prevalent chronic inflammatory bowel diseases that result from decreased immune responses to the antigens present in the gastrointestinal tract 29. Vitamin D has the potential to regulate gastrointestinal inflammation by influencing the gut microbiome. It acts as an adjunctive treatment therapy for inflammatory bowel disease due to its ability to regulate gut homeostasis, epithelial cells integrity, innate immune system, and the gut microbiome 30-33.

Wang *et al.* demonstrated an association between vitamin D deficiency and inflammatory bowel diseases. The mechanism involved a direct stimulatory effect of vitamin D on the nucleotide-binding oligomerization domain containing 2 (NOD2), which acts is major contributor to the diseases 34. Vitamin-D deficiency due to obesity could dysregulate gut homeostasis, leading to alterations in the trimethylamine-N-oxide (TMAO) pathway 35, 36, making it one of the risk factors for COVID-19.

Many genes expressing colon-cancer proteins are involved in regulating cell proliferation, differentiation, and apoptosis and contain elements of the vitamin-D response in the colon 37. Several lines of evidence show an inverse relationship between dietary vitamin-D3 intake or sunlight exposure with the incidence of polyps and adenomas in the colon, as well as human colorectal cancer. This highlights the key role of vitamin D in intestines 34.

#### Role of vitamin D in the intestine of individuals with COVID-19

COVID-19 infection is reported to cause gastrointestinal symptoms like diarrhea, vomiting, or abdominal pain during the disease and recovery, leading to inflammatory cytokine production 38. Acid reflux and stomach cramps are some of the striking symptoms during the second wave of COVID-19 infection, which are linked to mutated features of the virus. The most critical explanation of the gastrointestinal problems after COVID-19 infection was found to be the presence of angiotensin converting enzyme-2 (ACE-2) receptors in the digestive tract. These receptors enable the spike protein of coronavirus to bind with ACE-2 inhibitors and cause gastrointestinal problems 39. Therefore, for early diagnosis and effective treatment, it is important to detect gastrointestinal disorders following respiratory problems in individuals with COVID-19.

Vitamin D regulates the expression of ACE-2, which is the major target of the coronavirus. Vitamin D negatively regulates renin expression and interacts with the RAS/ACE/ACE-2 signaling axis, thus acting on the renin-angiotensin system. Vitamin D also regulates the gut microbiome and microbial diversity and favors gut-friendly commensal strains of *Bifidobacterium* and Firmicutes species 40. However, vitamin D deficiency results in the upregulation of RAS/ACE signaling and pro-inflammatory activity, which increases the incidence and severity of COVID-19 sepsis with manifestation of inflammatory bowel disease 41. The role of vitamin D in normal intestines and intestines affected by post-COVID-19 is shown in Table 1.

### Vitamin D and the pancreas

The rate of pancreatic diseases has been alarming in recent years and has become highly dangerous for patients and the public health system 42. Acute pancreatitis is associated with local and systemic inflammatory response syndrome due to increased inflammatory markers in the pancreas. In addition, pancreatic cancer has poor clinical outcomes with a 5-year survival rate less than 6%, ranking fourth in terms of cancer deaths in the Western world 43.

Single nucleotide polymorphism (SNP) in vitamin-D-related genes is associated with *CaSR*, a pancreatic cancer-risk gene, suggesting a strong correlation between vitamin D and pancreatic cancer 44. Hummel *et al.* reported that the expression of CYP24A1, a vitamin-D-degrading enzyme, is significantly enhanced during inflammation and malignant transformation, which has also been reported in chronic pancreatitis and pancreatic ductal adenocarcinoma 45. Upregulation of *CYP24A1* gene expression results in the degradation of 1,25(OH)2D3, thus attenuating the anti-tumorigenic action of vitamin D in tumor tissue.

#### Role of Vitamin-D the pancreas of individual with COVID-19

As discussed earlier, the entry of coronavirus into the host cells occurs upon binding of its spike protein with ACE-2. Recently, ACE-2 expression levels were reported to be higher in both the exocrine gland and the islets of pancreas than in the lungs of patients with COVID-19 post infection 46. Meireles *et al.* has reported a calculous pancreatitis on day 11 post COVID-19 infection in patients with pneumonia 47.

Easty *et al.* have explored the pathophysiology of Vitamin-D with special emphasis on COVID-19 and pancreatic cancer 48. In this study, Vitamin-D super agonists were employed, in order to limit the hypercalcemicactions and to enhance its efficacy on other beneficial effects. Vitamin-D, in this case, acts as a double-edged sword, where it acts as anti-fibrotic agent, which is helpful for patients with COVID-19, as well as, it works as anti-inflammatory agent to reduce COVID-19-induced inflammatory cytokines in COVID-19 patients. Nevertheless, individuals with Vitamin-D deficiency revealed greater susceptibility to infection as well as severity and mortality due to COVID-19 49. Further, the selective Vitamin-D superagonists showed to inhibit thecytokine (IL-6) release by regulatory T cells (Tregs) and that might reveal a promising effect on pancreatic cancer patients by reducing inflammation and desmoplasia 48, 50.

**Figure 2:**
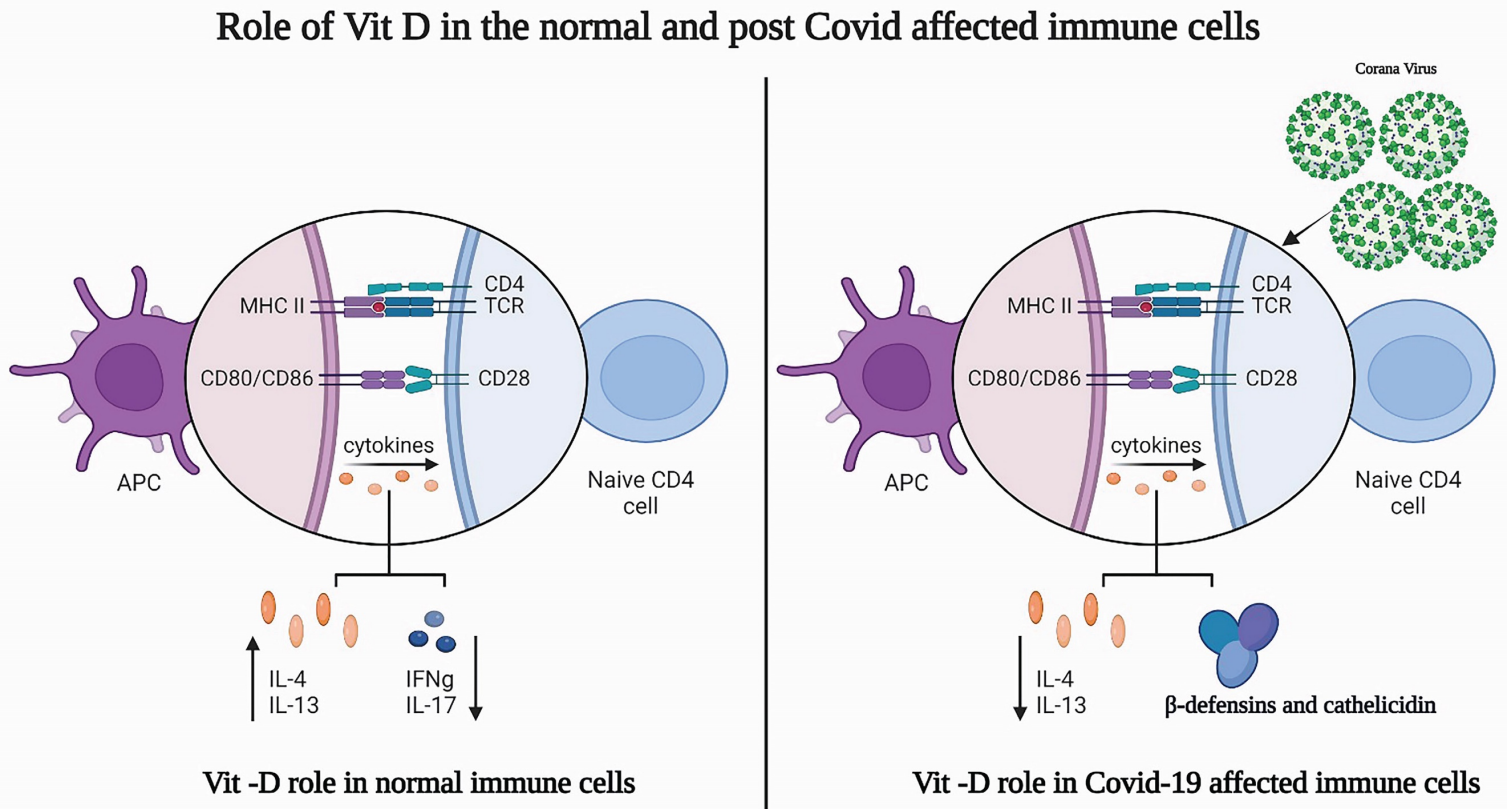
Role of vitamin-D in normal and post COVID-19 affected patient's liver and pancreas. a) Vitamin-D role in normal liver and pancreas b) Vitamin-D role in post COVID-19 affected patient's liver and pancreas. Image created from Bioreder.com.

The role of Vitamin-D in normal pancreas and post-COVID-19 affected pancreas is highlighted in Figure 2.

### Vitamin-D and the prostate gland

Many studies reported that the active form of Vitamin-D was able to regulate the immunity associated gene expressions in the prostatic tissue 51. De Marzo *et al.* suggested a mechanism of inflammation as one of the underlying reasons behind the pathogenesis of prostate gland cancer 52. In line with this, expression of various immune related genes was observed in prostate cancer, and particularly, significant variations in the gene expression were observed between black and white people 53. The report by Schwartz and Hulka was the first to demonstrate that an inverse relationship between serum Vitamin-D and the risk of prostate cancer 54. Vitamin-D was proposed to inhibit the proliferation of the tumor cells in prostate glands via mechanisms including cell cycle arrest, induction of apoptosis, and inhibition of the growth of the prostate epithelial cells in the glands 6. Importantly, Vitamin-D was reported to decrease invasion and adhesion of androgen-independent prostate cancer, in addition to its anti-proliferative effect 56.

In the presence of androgen, Vitamin-D significantly prevented the growth and the proliferation of human prostate cancer cell line, as compared to vitamin-D, suggesting the potential of the active form of Vitamin-D to induce prostate specific antigen release from LNCaP cells 57.

#### Role of Vitamin-D in prostate gland of individuals with COVID-19

Aaron *et al.* has studied whether Vitamin-D deficiency have any impact on the risk of COVID-19 among the breast and prostate cancer patients 58. A retrospective cohort analysis was adopted using nationally representative electronic medical records (EMR) to evaluate the role and the impact of Vitamin-D on prostate gland of COVID-19 affected patients. Their findings revealed that the COVID-19 risk was elevated among Vitamin-D deficient patients as compared to the Vitamin-D non-deficient patients in prostate gland cohort. Similar observations were documented in patients with newly diagnosed cancer in the dataset, supporting the fact that prostate cancer patients might have an alarming risk of COVID-19 infection upon Vitamin-D deficiency. In addition, patients with poor or less physical work/performance status due to certain treatments for any diseases displayed remarkable Vitamin-D deficiency coupled with higher susceptibility to COVID-19 infection 58. The study concluded, that Vitamin-D might have a potential to elicit a crucial role in other viral illnesses.

In general cancer patients are more susceptible to infection due to the immunocompromising nature of chemotherapies. Additionally, recent studies have suggested a significant risk for COVID-19 infection and hence poor prognosis in cancer patients 59.

The role of Vitamin-D in normal prostate and post-COVID-19 affected prostate gland is highlighted in Table 1.

### Vitamin D and the lung

Several studies suggested a significant role of Vitamin-D in lung immunity by influencing all the three innate immune effectors 60. Pretreatment of dendritic cells with Vitamin-D and CD4+ cells in co-culture, resulted in an induction of CD4+FoxP3+ (forkhead winged transcription factor) TRegs with reduced activity 61. Production of IL-10 secreting TReg population upon the interaction of Vitamin-D3 with CD4+ T cells was also found as an important mechanism behind the role of Vitamin-D in asthma 62. In a study by Schellenberg *et al.* a single nucleotide polymorphism of the Vitamin-D binding protein was associated with reduced chronic obstructive lung disease (COPD), and it found to influence the level of circulating 25-(OH)D3 and 1,25-(OH)2D3 63. Taq polymorphism of the VDR gene was reported to be one of the risk factors of lung cancer. Mernitz *et al.* has reported that Vitamin-D3 significantly reduced the growth of lung cancer in animal models 64.

#### Role of Vitamin-D in the lung of individuals with COVID-19

Various clinical parameters such as respiratory and biochemical parameters including 25OH-vitamin-D levels were assessed in COVID-19 patients by Sulli *et al.* 65. Vitamin-D levels in the serum of COVID-19 patients were significantly reduced as compared to normal subjects. Significant correlation was reported between the serum Vitamin-D levels and PaO2, SO2, PaO2/FiO2 levels. In contrast, a negative correlation was observed between serum vitamin-D levels and D-dimer, C-reactive protein, and percentage of oxygen. Further, an inverse correlation was identified between the worseness of radiologic pulmonary effect and Vitamin-D levels 65. COVID-19 patients with multiple lung consolidations or severe interstitial lung involvement were reported to be highly related to Vitamin-D deficiency. Interestingly, elderly COVID-19 patients, who lost their lives during hospitalization, displayed decreased serum Vitamin-D levels than the survivors. However, a remarkable decrease in Vitamin-D levels was also reported in young COVID-19 patients, suggesting Vitamin-D deficiency as a key risk factor associated with any age.

Murai *et al.* showed a positive correlation between 25-OH D and the clinical outcomes of respiratory diseases in COVID-19 patients 66. Regular dosage of Vitamin-D3 administration in elderly COVID-19 patients before the infection revealed better survival and less disease severity 67. However, no significant beneficial effect was observed when a single dose of about 200,000 IU of Vitamin-D3 was administered to the hospitalized patients with less to severe COVID-19 infection, thus signifying the importance of Vitamin-D in protection of the lungs in COVID-19 patients 68.

The role of Vitamin-D in normal and post-COVID-19 affected lungs is highlighted in Table 1.

### Vitamin D and the brain

As a neurosteroid, vitamin D increases the plasma β-amyloid protein in elderly people, which increases β-amyloid deposition 69. Vitamin D regulates the release of nerve growth factor (NGF), which is one of the essential molecules for the survival of hippocampal neurons and cortical neurons and is also known to reduce the risk of psychosis in children with chromosome 22q11.2 deletion 70. Gezen *et al.* identified a specific VDR-gene haplotype that is associated with an increased risk of Alzheimer's disease for the first time. Our investigations have also demonstrated that vitamin D protects against beta amyloid-induced calcium elevation and toxicity in cortical neurons, which are critical for NGF release.

Importantly, beta amyloid reduces VDR expression, and disruption of the vitamin-D-VDR pathway mirrors the neurodegenerative effects induced by beta amyloid 71. Several alterations in the brain, such as certain regionally selective neurotransmitter changes (dopamine/serotonin) and widespread neurotransmitter changes (glutamine/noradrenaline), were found to be correlated with vitamin D deficiency 72. In aging rats, vitamin D supplementation influenced pathways related to the upregulation of synaptic transmission, cell communication, and G-protein, thus bringing about a positive effect on learning and memory, reducing cognitive decline, and enhancing the likelihood of successful brain aging 73.

#### Vitamin D in the brains of individuals with COVID-19

Ceolin *et al.* 74 reported a close relationship between COVID-19 and mental health issues, such as a significant increase in the onset of depressive psychopathology and suicidal tendencies coupled with vitamin D deficiency. Binding of vitamin D to VDR stimulates the expression of tryptophan hydroxylase 2 (TPH2), serotonin reuptake transporter, and the levels of monoamine oxidase (the enzyme responsible for serotonin catabolism). Thus, it could influence the development of depressive symptoms in relation to altered light-dark cycles 74.

Vitamin D stimulates sustainable psychiatric symptoms and is expected to prevent psychiatric manifestations in individuals with COVID-19 75. Interestingly, an observational study carried out in Rome has reported a significant increase in psychological distress with mood disorders and depression in individuals with COVID-19 and lower serum levels of 25(OH)D 76. Depression is also found to be associated with mitochondrial dysfunctions leading to the formation of reactive oxygen species, which triggers the expression of several transcription factors associated with inflammation, in individuals with COVID-19 77. The role of vitamin D in the brain in normal conditions and after COVID-19 infection brain is shown in Table 1.

### Vitamin D and the liver

Vitamin D's role in the liver is supported by the expression of VDR in hepatic stellate cells, sinusoidal endothelial cells, and Kupffer cells upon inflammation 78. Vitamin D prevents the replication of hepatitis C viral RNA at higher concentrations than that observed in the circulation. This occurs through action on interferons and enhanced autophagic genes, such as G-protein coupled receptor-37 79. Ding *et al.* showed that ligation of VDR in activated hepatic stellate cells leads to anti-fibrotic effects that are mediated through a VDR/SMAD3/TGF-β signaling loop, suggesting that vitamin D inhibits liver fibrosis 80. VDR agonist and calcipotriol were found to improve liver function, decrease liver inflammation, necrosis, and the fibrosis percentage. It also led to a reduction of hepatic collagen-1α, a tissue inhibitor of metalloproteinase, TGF-β1 protein, and activity of the TGF-β-SMAD pathway 81.

#### Role of vitamin D in the livers of individuals with COVID-19

Research has reported various types of liver injuries in individuals with COVID-19 82. In many cases, vitamin D deficiency provokes vulnerability to acute viral respiratory infections. Therefore, administration of vitamin D was found to boost the innate immune responses to influenza and viral hepatitis 83. Furthermore, vitamin D might have a beneficial role in liver tissue via antioxidant and anti-inflammatory effects 84. Importantly, Meltzer *et al.* investigated the changes in vitamin D status between before COVID-19 testing and the occurrence of COVID-19 positivity with an emphasis on various parameters, including liver diseases 85. The study identified an association between vitamin D deficiency and possibly inadequate recovery from COVID-19. The role of vitamin D in the liver in normal conditions and after COVID-19 infection is shown in Figure 2.

### Vitamin D and the kidneys

Studies show that vitamin D has a role in targeting several pathways in the kidneys. In particular, a key specific pathway that is regulated by vitamin D is the renin-angiotensin system during chronic kidney diseases. Higher VDR activity has been shown to reduce the deleterious effects of chronic kidney diseases, such as hypertension, cardiac hypertrophy, and increased water intake, along with reductions in glomerular and tubulointerstitial destruction 86, 87. Vitamin D plays a very important role in acute kidney injury by reducing oxidative stress damage, proteinuria, damage in the podocytes, macrophage infiltration, dilation of mesangial cells, proinflammatory profibrogenic factors, extracellular matrix proteins, and neutral lipid accumulation 87. Vitamin D also reduces the onset of inflammation and myofibroblast production through down-regulation of the expression of TLR-4 and MCP-1, thus exerting protective effects on the kidneys 88.

#### Role of vitamin D in the kidneys of individuals with COVID-19

It has been reported that most individuals with COVID-19 had preexisting complications, including cardiovascular, respiratory, and renal disorders. They also show significantly abnormal markers such as vitamin D deficiency, reduced glomerular filtration rate, and interleukin-6. Lower levels of vitamin D are associated with reduced kidney function reflected by a lower glomerular filtration rate during the manifestations of COVID-19 89. Generally, individuals with COVID-19 more commonly have a long-term clinical history of kidney or heart disorders, which is supported by a disordered renin-angiotensin system.

The most common manifestation in individuals with COVID-19 is the prevention of angiotensin II accumulation through inhibition of renin release by vitamin D, which acts as a functional inhibitor of the renin angiotensin system 90. Furthermore, angiotensin-II was found to increase the uncontrolled cholesterol plaque formation along the vessels and podocytes, which in turn provokes systemic glomerular hypertension and leads to ischemic-induced kidney injuries, ultimately resulting in kidney failure. Vitamin D deficiency in the kidneys is considered as a critical factor in COVID-19 in relation to significant inflammation and kidney failure 3, 91. Moreover, uncontrolled increase of interleukin-6 in association with a reduced glomerular filtration rate might correlate with a state of immune complications due to vitamin D deficiency 92. The role of vitamin D in the kidneys in normal conditions and after COVID-19 infection is shown in Table 1.

### Vitamin D and the heart

Several studies have demonstrated an association of vitamin D with cardiac protection and suggested its potential therapeutic benefits. Vitamin D deficiency correlates with high risk of cardiovascular diseases such as coronary artery disease, hypertrophy, myocardial infarction, fibrosis, cardiomyopathy, and heart failure 93. Vitamin D deficiency was found to be associated with many arterial diseases such as aneurysm, arterial calcification, peripheral arterial disease, hypertension, and atherosclerosis. Furthermore, cardiac inflammation was observed to be associated with vitamin D deficiency along with oxidative stress, energetic metabolic alterations, cardiac hypertrophy, changes in the left auricle and ventricle, systolic dysfunction, fibrosis, and apoptosis 94.

The modulation of ST2, a receptor that binds with IL-33 and regulates cardiac function, is also known to be modulated by vitamin D 95. Oz *et al.* correlated vitamin D deficiency with slow coronary flow, dysfunctions in endothelial cells, and atherosclerosis 96. Vitamin D has anti-inflammatory, anti-apoptotic, and anti-fibrotic mechanisms that enhance cardio protection.

#### Role of vitamin D in the hearts of individuals with COVID-19

Vitamin D plays a role in various cardiovascular conditions that may exacerbate the severity of COVID-19 infection, such as hypertension, lipid metabolism disorders, atherosclerosis, and heart failure 97. An aggressive inflammatory response leading to hypercoagulability has been linked to disease severity in individuals with COVID-19, which negatively impacts treatment outcomes 98. Critically ill patients in ICUs were found to show signs of acute disseminated intravascular coagulation (DIC), as well as pulmonary embolism and deep vein thrombosis. Due to the immunomodulatory effects of vitamin D on immune cells, vitamin D supplementation is recommended worldwide to alleviate clinical symptoms for individuals with COVID-19. Additionally, vitamin D and its associated molecules are known to regulate various thrombotic pathways either directly or indirectly. Therefore, it has been demonstrated that vitamin D supplementation may not only reduce the risk of acute respiratory distress syndrome (ARDS), but also potentially mitigate coagulation abnormalities in critically ill individuals with COVID-19 98.

The body's immune response to SARS-CoV-2 infection leads to COVID-19, and varying levels of severity are often linked to an exaggerated inflammatory reaction. This excessive response is known as a "cytokine storm" and involves a significant and prolonged release of pro-inflammatory cytokines that contribute to symptoms and organ damage, particularly in the lungs and heart 99. Studies indicate that in severe cases of COVID-19, IL-6 levels can be nearly three times higher than in milder cases. Vitamin D has been shown to play a very important role in mitigating cytokine storms by promoting the production of anti-inflammatory agents like IL-10, IL-4, and TGFβ. Additionally, vitamin D helps in reducing hyperinflammation by favoring anti-inflammatory and regulatory immune responses (Th2 and T-reg) over the pro-inflammatory responses (Th1/Th17) that are more prominently involved in cytokine storms, thus reducing the organ damage 99.

Vitamin D influences endothelial cell function, thereby regulating vasodilation in endothelial-dependent pathways. Vitamin D supplementation can potentially prevent atherosclerosis and vascular calcification, which are conditions that increase the risk among individuals with COVID-19. Additionally, vitamin D reduces pro-inflammatory cytokines, potentially mitigating the risk of severe outcomes like obesity and heart failure among individuals with COVID-19 100. The role of vitamin D in the heart in normal conditions and after COVID-19 infection is shown in Table 1.

Importantly, VDR has also been found to play a very important role in individuals with COVID-19 in addition to vitamin D. There is growing interest in understanding how VDR and its expression may influence COVID-19 outcomes. While much attention has been given to vitamin D itself, several studies have explored irregular expressions of VDRs and their effect on the immune response and the severity of COVID-19 101-103. The relationship between VDR SNPs and COVID-19 symptoms may differ based on the severity of the disease.

A study comparing VDR polymorphisms between patients with severe and mild forms of COVID-19 showed that the TT genotype of the rs11568820 polymorphism appears to have a protective effect by reducing the risk of severe disease and hospitalization 101. Vitamin D deficiency and the VDR Fok I polymorphism have been found to serve as independent risk factors for increased susceptibility to COVID-19 among children and adolescents in Egypt 102, 104. VDR has also been reported to play a very important role in the pathophysiology of ARDS. These findings demonstrate the significant role of vitamin D and VDR in COVID-19.

## Conclusion

This review has summarized the beneficial effects of vitamin D in several organs in normal conditions and after COVID-19 infection. The results illustrate the vital need for vitamin D in daily life. This review has also highlighted the advantages of vitamin D as an ideal and promising therapeutic strategy for various disorders, in addition to its immune-boosting action. The role of VDR in COVID-19 infection was also emphasized. However, issues regarding the appropriate dose, duration, and mode of administration of vitamin D remain unanswered and require further research. In-depth clinical studies are needed to identify the role of vitamin D in combatting diseases such as COVID-19.

## Figures and Tables

**Table 1:** Role of vitamin-D in normal and post COVID 19 affected conditions in intestine, prostate gland, lungs, brain, kidney and heart.

ORGANS	VITAMIN-D ROLE IN NORMAL CONDITIONS	VITAMIN-D ROLE IN COVID-19 AFFECTED CONDITIONS	REFERENCES
INTESTINE	1. Regulates inflammation through gut microbiome. 2. Regulates transcription of cathelicidin and DEFB4. 3. Decreases polyp recurrence in colon cancer patients.	1. Interacts with RAS/ACE/ACE-2 signaling axis and regulates rennin-angiotensin system. 2. Regulates gut microbiome and microbial diversity. 3. Reduces inflammatory bowel diseases.	33, 34, 40, 41
PROSTATE GLAND	1. Reduces inflammation in prostate gland cancer. 2. Inhibits proliferation of tumor cells. 3. Enhances cell cycle arrest and apoptosis. 4. Inhibits growth of prostate epithelial cells. 5. Reduces invasion and adhesion of androgen-independent prostate cancer. 6. Increases anti-metastatic potential.	1. Reduces severity of Coronavirus in prostate cancer patients. 2. Enhances anti-metastatic potential in COVID-19 affected prostate cancer patients.	52, 54, 56, 57, 58, 59
LUNGS	1. Regulates innate immune effectors, airline epithelium, alveolar macrophages and dendritic cells. 2. Enhancescathelicidin secretion. 3.Downregulateschemokines production. 4. Prevents dendritic cell activation. 5. Regulates T cell activation. 6. Enhances immunity through CD4+FoxP3+ Tregs expression. 7. Prevents asthma through increasing IL-10 secreting TReg population. 8. Reduces lung cancer growth.	1. Increases PaO2, SO2, PaO2/FiO2 levels. 2. Reduces severity in COVID-19 patients with multiple lung consolidations or severe interstitial lung involvement. 3. Decreases mortality of hospitalized elderly COVID-19 patients.	60-67
BRAIN	1.Regulates β-amyloid deposition in older adults. 2. Regulates calcium levels, decreases the onset of depression. 3. Regulates nerve growth factor release. 4. Decreases risk of psychosis in children with chromosome 22q11.2 deletion. 5. Upregulates synaptic transmission, cell communication, and G-protein. 6. Enhances learning and memory. 7. Reduces cognitive decline.	1. Upregulates serotonergic neurotransmission during depression of COVID-19 patients. 2. Increases expression of tryptophan hydroxylase 2 and monoamine oxidase. 3. Decreases psychological distress with mood disorders in the COVID-19 patients. 4. Regulates sleep-wake cycle. 5. Prevents inflammatory disorders associated with depression.	69-77
KIDNEY	1. Regulates renin-angiotensin system. 2. Decreases hypertension, cardiac hypertrophy and increased water intake. 3. Reduces glomerular and tubulointerstitial destruction. 4. Inhibits proteinuria. 5. Prevents activation of NF-κB. 6. Decreases oxidative stress damage in the podocytes, macrophage infiltration, dilation of mesangial cells, andproinflammatoryprofibrogenic factors, extracellular matrix protein. 7. Reduces neutral lipid accumulation. Decreases inflammation and myofibroblasts production by downregulating TLR-4 and MCP-1.	1. Upregulates glomerular filtration rate in COVID-19 patients. 2. Inhibits angiotensin-II induced uncontrolled cholesterol plaque formation. 3. Prevents angiotensin-II within the myocardium and renal cortex. 4.Downregulates IL-6 production.	86-92
HEART	1. Inhibits aneurysm, arterial calcification. 2. Prevents peripheral arterial disease, hypertension, and atherosclerosis. 3. Regulates coronary flow. 4. Reduces inflammation, oxidative stress, and energetic metabolic alterations. 5. Decreases cardiac hypertrophy, changes in the left auricle, and ventricle. 6. Decreases systolic dysfunction, fibrosis, and apoptosis. 7. Regulates ST2 levels. 8. Regulates renin angiotensin-aldosterone system. 9. Decreases blood pressure, hypertrophy, fibrosis and thrombosis. 10. Regulates cardiovascular risk factors.	1. Mitigates coagulation abnormalities in crtically ill COVID-19 patients 2. Prevents cytokine storms by stimulating anti-inflammatory agent. 2. Influences endothelial cell function and regulates vasodilation 3. Prevent atherosclerosis and vascular calcification in COVID-19 patients.	93-100
